# Maximizing influenza vaccination uptake among healthcare personnel in Israel: lessons learned from mandatory vaccination policy in the United States

**DOI:** 10.1186/s13584-019-0326-4

**Published:** 2019-09-16

**Authors:** Rachel Gur-Arie

**Affiliations:** Department of Health Systems Management, School of Public Health, Faculty of Health Sciences, Ben-Gurion University of the Negev, Beersheba, Israel

**Keywords:** Healthcare personnel, Influenza vaccination, Mandatory vaccination, Public health policy, Ethics

## Abstract

**Background:**

Maximizing vaccination uptake is crucial in generating herd immunity and preventing infection incidence (Quach et al., Am J Infect Control 11:1017–23, 2013). Vaccination of healthcare personnel (HCP) against influenza is vital to influenza infection control in healthcare settings, given the consistent exposure of HCP to high-risk patients like: those with compromised immune systems, children, and the elderly (Johnson & Talbot, Curr Opin Infect Dis 24: 363–369, 2011). Influenza vaccination uptake among HCP remains suboptimal: in 2017–18, 47.6% of HCP who worked in settings where influenza vaccination was not mandatory were vaccinated against influenza in United States (Black et al., Morb Mortal Wkly Rep 67: 1050, 2018). Mandatory vaccination policies result in HCP influenza vaccination uptake rates substantially higher than opt-in influenza vaccination campaigns (94.8% vs. 47.6%) (Black et al., Morb Mortal Wkly Rep 67: 1050, 2018).

**Goals:**

The Israel Journal of Health Policy Research has published articles focused on the issues of influenza vaccination (Yamin et al., Isr J Health Policy Res 3: 13, 2014), improving influenza vaccination coverage of HCP (Weber et al., Isr J Health Policy Res 5: 1–5, 2016), influenza vaccination motivators among HCP (Nutman and Yoeli, Isr J Health Policy Res 5: 52, 2016), legal imposition of vaccination (Kamin-Friedman, Isr J Health Policy Res 6:58, 2017), and mandatory vaccination (Gostin, Cell Biosci 8: 1-4, 2018). Each article explores factors influencing disease prevention from different angles within an Israeli context. This article attempts to fuse these topics by investigating how to apply aspects of American mandatory influenza vaccination policy targeted at HCP in an Israeli context.

**Methods:**

Critical document analysis was conducted on relevant literature and policy discussing influenza prevention interventions among HCP within the United States. Mandatory vaccination policies were highlighted. A discussion of the professional responsibility of HCP to vaccinate against influenza serves as background. Case studies of hospitals in the United States that implemented mandatory vaccination policies for their employees are analyzed. The article concludes with analysis exploring how qualities of mandatory influenza vaccination policy of HCP could take shape in Israel, giving contextual limitations, urging Israeli health policy makers to reflect on lessons learned from the American case study.

**Main findings and conclusion:**

Mandatory HCP influenza vaccination policies in comparison to non-mandatory interventions are most effective in obtaining maximum influenza vaccination uptake among HCP (Black et al., Morb Mortal Wkly Rep 67: 1050, 2018). Many HCP cite individual objections to influenza vaccination rooted in personal doubts and ethical concerns. The ethical responsibility of HCP to their patients and work environments to prevent and lower influenza infection incidence arguably overrules such individual objections. Mandatory HCP influenza vaccination policies are an effective method of maximizing HCP influenza vaccine uptake and minimizing the spread of the influenza virus within healthcare settings. Still, cultural, social and political sensitivity must be taken into consideration when implementing both full-on mandatory HCP influenza vaccination policies and/or aspects of mandatory policies, especially within an Israeli context.

## Background

### The threat of influenza to healthcare personnel (HCP)

Influenza is a substantial, present threat to healthcare settings [6, 21]. Influenza outbreaks in long-term care facilities are frequent, occurring in as many as 50% of facilities [18]. HCP include physicians, nurses, physician and nursing assistants, technicians, emergency medical service personnel, dental personnel, pharmacists, laboratory personnel, and students. HCP are believed to be at increased risk of influenza infection [18] due to their regular exposure to populations most vulnerable to influenza contraction: the elderly, youth, and patients with underlying medical conditions [4]. A meta-analysis of studies of seasonal influenza among HCP estimated that on average, about 1 in 5 HCP get sick with influenza and are symptomatic each each [18]. Less than half of influenza virus infections are symptomatic [10], and HCP often engage in presenteeism (working while ill) [31], which further increases the risk of transmitting respiratory viruses to vulnerable patients [15].

### HCP and the influenza vaccine

Influenza vaccinations are the most effective nosocomial influenza prevention intervention among HCP when compared to other prevention methods [6]. Higher vaccination rates among HCP are associated with lower incidence of nosocomial influenza cases [6]. Large numbers of unvaccinated HCP allows influenza to propagate quicker, faster, and in increased severity [18]. Influenza among HCP can spread via nosocomial infection as early as one day prior to symptomatic illness and as late as five to 10 days post-symptomatic illness [19]. However, on average, only half of HCP show classic symptoms for influenza, challenging influenza prevention and control interventions [19].

The United States and Israel both generally promote influenza vaccination among HCP [3, 12]. Israel is considered to be a “highly vaccinated society”, with vaccination uptake over 90% among the general public for many vaccinations not mandated by Israeli law [2]. Nevertheless, influenza vaccination uptake rates of HCP in both the United States and Israel are consistently suboptimal [3, 6]. In 2017–18, 47.6% of HCP who worked in settings where influenza vaccination was not mandatory were vaccinated against influenza in the United States [6]. According to the Israeli Ministry of Health, 24% of HCP were vaccinated against influenza during the 2014–2015 influenza season [3].

### The professional responsibility of HCP to vaccinate against influenza

The professional duties of HCP include competence, honesty with patients, patient confidentiality, maintaining appropriate relationships with patients, improving quality of care, just distribution of finite resources, maintaining modern scientific knowledge, and managing conflicts of interest [30]. These responsibilities are separate from the professional values taught in healthcare and medical curricula, including altruism, respect for others, honor, integrity, ethical and moral standards, accountability, excellence, and duty/advocacy [32]. Nevertheless, there are clashing viewpoints regarding how much emphasis should be placed on each individual responsibility and value – if at all. Sometimes, medical educators include the values of autonomy, self-regulation, and dealing with uncertainty [25], while others discard these concepts altogether [30].

In spite of the notion that harm (influenza) may occur if no preventative action (influenza vaccination) is taken [34], vaccination rates for HCP are suboptimal when not mandatory [6], despite many institutions, like the Centers for Disease Control and Prevention (CDC) in the United States, recommending annual seasonal influenza vaccination of HCP [8]. The education of HCP in infection control in combination with regular institutional recommendations raises the questions of why HCP influenza vaccination uptake rates are consistently inadequate, as well as how to improve them. This paper explores the feasibility of applying aspects of mandatory policy approaches based on examples set in the United States to influenza prevention interventions among HCP in Israel.

## Methods

Critical document analysis was conducted on relevant literature and policy discussing influenza prevention interventions among HCP within the United States, with a focus on mandatory vaccination policies. A discussion of HCP professional responsibility to vaccinate against influenza precedes such analysis. Case studies of hospitals in the United States that implemented mandatory vaccination policies for their employees are explored. The article concludes with analyzing how aspects of HCP mandatory influenza vaccination policy could be applied in Israel, urging health policy makers to reflect on American case studies.

## The professional responsibility of HCP to vaccinate against influenza

Mandatory influenza vaccination of HCP can be ethically justifiable based on four key principles: (1) the professional duty to prioritize patients’ interests above all else, (2) the obligation to ‘do no harm’, (3) the requirement to protect those who cannot protect themselves; and (4) the obligation to set a good example for the public [9]. Beneficence, non-maleficence, and justice are guiding principles of medical practice [28]. All HCP are expected to uphold the core medical ethicof “First Do Not Harm”. The same obligation applies to HCP employers (healthcare and medical-providing institutions) in order to establish a workplace culture of promoting influenza vaccination. The viewpoints of both employers (healthcare and medical settings) and employees (HCP) contribute to the multi-layered complexity of the ethical debate surrounding HCP mandatory influenza vaccination policies.

Patients expect that healthcare facilities and HCP take “reasonable measures to ensure that their care is as safe as possible (non-maleficence)” [28]. Under this assumption, HCP take all reasonable measures to prevent the transmission of communicable, infectious diseases such as influenza [28]. Tilburt et al. suggests that preferable prevention methods exist in the form of safe, effective vaccines [28]. However, counterarguments may suggest other influenza-prevention methods are equally sufficient in fulfilling expected “reasonable measures” [28]. Even still, the majority of ethical appeals to HCP mandatory influenza vaccination policies are rooted in claims of personal autonomy and right-to-choice [22]. This leads to the question of whether HCP perceptions of influenza vaccination translate into action (getting vaccinated), or inaction (not getting vaccinated) which uphold their professional “duty” to patients [22].

Anti-mandatory vaccination arguments are rooted in claims of personal autonomy infringement and professional responsibility. Mandatory influenza vaccination policies are employment-contingent policies which generally maintain courtesy towards HCP autonomy via medical and religious exemptions. Autonomy, defined as acknowledging a person’s right to make choices and decisions [19], is one of many moral considerations that must be weighed in when ethically evaluating mandatory influenza vaccination policies targeted at HCP. Anti-mandatory vaccination stances rooted in concerns regarding professional responsibility appeal to private choice. Such claims do not view vaccination as a justifiable required action based on the professional duties of HCP, claiming that it intrudes on private (mental and bodily) rights [29].

Alternative non-mandatory vaccination policies targeted at HCP usually take form in opt-out vaccination policies which are implemented via declination forms. If HCP do not wish to be vaccinated, they “opt-out”, and their appeal to personal autonomy in refusing vaccination is respected [19]. A major consequence of this “softer” policy, in comparison to mandatory vaccination, is that HCPs’ compliance to influenza vaccination is largely unpredictable and varied at best. Vulnerable patients are not maximally protected against harm (influenza) [19]. Perhaps a policy that implements restricted mandatory vaccination in addition to opt-out declination forms could offset sub-par vaccination uptake resulting from opt-in policies [19]. When a compelling institutional threat of influenza is officially recognized, more attention is usually drawn to the lacking HCP influenza vaccination uptake achieved through opt-in programs. For this reason, among others, Tilburt et al. and Gostin argue that HCP mandatory influenza vaccination policies are ethically justifiable and merit implementation. Despite potential legal and ethical soundness, in certain countries, including Israel as of 2019, mandatory vaccination of HCP is not a feasible immediate policy decision thanks to a variety of political and systematic hurdles [17]. With this in mind, this paper emphasizes the importance of weighing social, cultural, and political environments before implementing mandatory vaccination policies. Additionally, even if mandatory policies are not implemented, certain successful characteristics can be applied to inventing creative alternative policies.

## The American case study

Current policy regarding HCP influenza vaccination in the United States is inconsistent. This is largely due to varying state governance and regulations usually in the form of recommendations [26]. For over 30 years, several governmental and non-governmental societies have consistently recommended HCP influenza vaccination [4]. Such organization is the Advisory Committee On Immunization Practices, which first recommended annual influenza vaccination of HCP in 1984 [4]. The Society for Healthcare Epidemiology, the Association for Professionals in Infection Control, and the Infectious Disease Society of America also heavily endorse influenza vaccination of HCP [4]. One of the *Healthy People 2020* goals is to achieve 90% influenza vaccination coverage among HCP in the United States [4]. Given that recommendation and encouragement from the employer, governmental organizations, and non-governmental institutions results in suboptimal influenza vaccination uptake among HCP, the *Healthy People 2020* goal bolstered debate on policies that can successfully and sustainably increase HCP influenza vaccination uptake [4].

### Opt-in influenza vaccination campaigns

Incentivized recommendations, in the form of workplace-implemented “opt-in” vaccination campaigns, provide influenza vaccination to HCP free of charge at their place of work [1]. Multi-faceted quality-improvement initiatives, usually in the form of educational and interaction-focused opt-in vaccination campaigns, have variable success within healthcare institutions in raising HCP vaccination rates above 60% [1]. Other healthcare-providing settings find it difficult and/or impossible to reach and maintain coverage above 75% [27]. Institutions utilizing opt-in campaigns have no power to enforce vaccination. Because HCP have to ‘opt-in’ to participate, they usually have to take time out of their work schedules, or cut into their personal time, to ultimately receive the influenza vaccine.

### Mandatory influenza vaccination

Mandatory influenza vaccination policies are employment-conditioned and effective in maximizing HCP influenza vaccination uptake. According to the Society for Healthcare Epidemiologists of America (SHEA), components of successful mandatory vaccination programs include: programmatic principles that allow the policy to be comprehensive and provide ready access to vaccination (inclusive to free vaccination), employing targeted education that emphasizes the rationale for a mandatory policy, a strong leadership commitment, and steady resources [7]. Mandatory vaccination policies geared toward HCP are more than black-and-white regulations that require influenza vaccination without accounting for initial or sustained rebuttal and/or objection. They incorporate diverse strategies that provide those medically unable to participate or the minority that personally refuse vaccination. Compromises include using vaccination rates as a measure of the facility’s safety and quality program, requiring unvaccinated HCP to wear a mask during influenza season, and using signed declination statements for HCP who refuse vaccination [7].

Even within institutions that do not enforce mandatory vaccination, consequences to vaccination refusal exist. These consequences, to which nonmedical exemptions are commonly accepted, include wearing a mask during work and terming/identifying unvaccinated HCP [20]. Additionally, when HCP sign declination forms to allow them to continue working without vaccination, the declination forms frequently remind the HCP of the risks of not being vaccinated, including both personal risk and risk of transmission to patients [20].

Virginia Mason Mason Medical Center (VMMC) in Seattle, Washington was the first healthcare setting in the United States to implement a mandatory influenza vaccination policy among HCP [23]. Suboptimal vaccination uptake in August 2004 prompted hospital decision-makers to implement a mandatory influenza vaccination policy, which extended to all non-VMMC employees working within the medical center, such as community physicians, vendors, students, and volunteers [23]. The initial policy, implemented in 2005, was extremely strict for a first-time mandatory HCP influenza vaccination policy. Declination statements and appeals, which are usually written into the mandatory policy as a way to maintain HCP autonomy and self-dignity, were not accepted from any HCP without medical justification [23]. While there was apparently initial resistance to the policy, no significant literature exists supporting this claim [23].

Since the implementation of the VMMC mandatory vaccination policy, influenza vaccination uptake of over 5000 HCP had been consistently sustained above 98% as of 2010 data [26]. Following the example of VMMC, multiple healthcare institutions across the United States implemented mandatory influenza vaccination policies targeted at HCP and have since sustained comparable success to that of VVMC. Such institutions include, but are not limited to: BJC Healthcare (Barnes-Jewish-Christian Healthcare) in St. Louis, Missouri; CHOP (Children’s Hospital of Philadelphia) in Philadelphia, Pennsylvania; HCA (Hospital Corporation of America) in Nashville, Tennessee; and MedStar Health in Columbia, Maryland [16]. A study of 1062 US hospitals found that according to the 2017 US National Survey, more than two-thirds of non-VA (Veteran's Health Administration) hospitals mandated HCP influenza vaccination [14].

### Case study: BJC healthcare (Barnes-Jewish-Christian healthcare)

Up until 2007, HCP influenza vaccination policy at BJC was promoted by annual opt-in influenza vaccination campaigns [4]. In 2007, influenza vaccination was added to the BJC patient safety and quality scorecard [4]. Hospital leaders were incentivized to raise HCP influenza vaccination uptake. Despite professional efforts by occupational health and infection prevention specialists, influenza vaccination uptake among HCP remained below the goal of 80% uptake [4]. In response, in 2008, BJC Healthcare implemented a mandatory influenza vaccination policy targeted at HCP [4].

Following the implementation of the mandatory influenza vaccination policy, of almost 26,000 active BHC HCP, 98.4% were vaccinated against influenza [4]. 1.24% were medically exempt and 0.35% were religiously exempt [4]. 99.96% of employees complied with policy regulations (vaccinated or exempt), with only 8 employees (0.03%) terminated for policy noncompliance [4]. 100% of BJC-employed physicians, including about 900 residents and fellows, received their influenza vaccination [4]. Most terminated HCP did not submit an exemption request. Only 21 HCP (0.08%) reported a possible adverse reaction to the influenza vaccine [4]. However, the majority of adverse reactions were unable to be objectively linked to the influenza vaccine due to many other potential antecedent triggers [4].

Within BJC’s plan, temporary (one year) or permanent medical or religious exemptions could be requested. Premedical condition exemptions, reviewed by occupational health nurses and their directors, included hypersensitivity to eggs, prior hypersensitivity reaction to the influenza vaccine, and a history of Guillain-Barre syndrome [4]. While unenforced, BJC administration encouraged exempted HCP to wear masks while caring for patients during the influenza season [4]. HCP who did not meet either medical or religious criterion for exemption were welcome to express concerns to BJC occupational health nurses and/or medical directors, but were not necessarily entitled to an exemption. [4].

Babcock et al.’s study illustrates the overwhelming efficacy of mandatory vaccination policies in consistently increasing HCP influenza vaccination uptake to over 90% [4]. The program was established as a patient safety initiative, and benefitted from strong leadership support, solid infrastructure, and timely and consistent communication between all parties involved [4]. For this reason, expecting similar success to that of BJC’s mandatory HCP influenza vaccination campaign should not be immediately assumed when applying its tactics to different settings.

## The Israeli case study

National and cultural specificity might be a way to point out how other non-medical influences inform how HCP think and act in different medical-social-legal-cultural environments. These similarities and differences are important both in the justification but perhaps more in the implementation of mandatory influenza vaccination policies directed at HCP.

This study uses Israel as a case study for cultural specificity regarding mandatory influenza vaccination policy of HCP. Yamin et al. suggests that socio-demographic and socio-economic diversity in the Israeli population may necessitate disease prevention interventions be customized to the preferences of sub-populations [36]. Even still, there are qualities that characterize specific societies and cultures. Results of a study of the willingness of Israeli HCP to risk their lives for patients during the peak of the 2009 influenza A H1N1 pandemic suggest that investing resources in increasing the safety of HCP significant increased the chances of HCP attending work during pandemic avian flu [5]. Trust in colleagues and HCP willingness to risk their lives for others suggest a correlation to the military maxim, “one for all and all for one”, when soldiers are willing to risk their lives for their peers because they know the behavior is reciprocal [5]. Applying this axiom to HCP decision-making is not out of the ordinary within an Israeli context. The influence of military and healthcare disaster preparedness and management is historically tied to the curriculum of HCP training in Israel [5].

While seasonal influenza vaccination for HCP is recommended by the Israeli Ministry of Health, it is not consistently regulated across Israel [3]. Sometimes, contradictory messages emerge. Deputy Health Minister Yaakov Litzman told the Jerusalem Post that influenza vaccines “apparently didn’t work” because he “got the flu” after being vaccinated [24]. According to the Israeli Ministry of Health Director General, it is the responsibility of medical administrations within workplaces to promote and regulate influenza vaccination among HCP. Naturally, this leads to diverse regulation, implementation, and ultimately influenza vaccination uptake among HCP across healthcare settings in Israel. Overall, the influenza vaccine remains largely optional for HCP.

Kamin-Friedman examined the legality of mandatory vaccination in Israel in light of the 2013 detection of polio in Israeli sewage. Though vaccinating children proved to be significantly more difficult than vaccinating HCP for polio, legal justification used for imposing polio vaccination on children in 2013 could be similar to that of mandatory influenza vaccination of HCP, if supportive socio-political circumstances arose in Israel. Kamin-Friedman suggests that mandatory polio vaccination supported by criminal sanctions would probably be perceived as infringing on the Israeli constitutional right to autonomy in a greater way than established by law and case precedents. Still, the Israeli Basic Law: Human Dignity and Liberty states that the government has an obligation to protect the life, body, and dignity of every individual [17].While the right to health has not been recognized as a basic right in Israel [17], the importance of eradicating infectious diseases to protect human dignity, life, and body could potentially uphold the feasibility of at minimum applying mandatory influenza vaccination policy to specific populations of HCP working with immunocompromised populations, the elderly, and children.

Value is found in new public health law and policy that authorizes public health officials (or healthcare institutions) to oblige vaccination when nonrestrictive measures (such as influenza vaccination recommendation and/or opt-in vaccination campaigns) are ineffective [17]. While government should always use the least invasive/restrictive alternative to achieve public health objectives, vaccination requires the large majority of a given population to be vaccinated in order to generate “herd” immunity [13]. Mandatory vaccination, according to Gostin, is well within the “harm principle” which justifies compulsion to prevent individuals from putting others at risk [13]. Applying this logic to influenza vaccination of HCP is sound given their professional duties and work setting. However, given strict laws protecting workers’ rights as of 2019, the feasibility of implementing mandatory influenza vaccination policy targeted at HCP in Israel is low [35].

Nevertheless, the non-feasibility of implementing mandatory influenza vaccination policy for HCP does not mean that there is no room for strengthening efforts targeted at influenza prevention in Israel. Within an Israeli context, this will most likely not take form in regulative measures. This leaves a large responsibility among healthcare administrators, managers, and bosses to create a culture around rewarding influenza vaccination and “reprimanding” those who do not choose to vaccinate for non-medical or non-religious reasons. Such “punishments” need not take shape in termination, which is prohibited anyway under Israeli law [35]. Employers can choose to hire only vaccinated HCP to work in certain departments, which complies with worker’ protection laws, since the HCP is employed, just perhaps not in their preferred department. Campaigns to wear “I vaccinated!” stickers or to promote wearing masks for those unvaccinated is another tool that could be used to generate stigma among unvaccinated HCP. However, generating stigma among HCP for choosing not to vaccinate against influenza may be less appealing within Israeli society, given the value and respect placed upon the individual right to choice. Still, it is a tactic worth exploring.

## Conclusions

Public health recognizes state power and responsibility to protect health and safety without overstepping [13]. In 2005, SHEA defined influenza vaccination of HCP as “a core patient and HCP safety practice with which noncompliance should not be tolerated” [26]. Healthcare facilities have responsibilities to take “reasonable measures” to ensure that influenza prevention interventions are as safe and effective as possible [28].

Cognizant of policy alternatives and respectful of professional ethical concerns, this article explores mandatory HCP influenza vaccination policies within the United States in order to extrapolate potential applications to an Israeli context. There is a time and place for coercive and persuasive influenza vaccination interventions [11]. Coercive policies regarding HCP and influenza vaccination raise HCP influenza vaccine uptake to levels that can generate herd immunity and lower influenza incidence in healthcare settings [33]. Case studies and lessons learned from the United States provide scientific and ethical support for the implementation of mandatory HCP influenza vaccination policies. Nevertheless, cultural, legal, political, and social sensitivities often take precedent, as is the case in Israel in 2019. Even still, successful characteristics of American mandatory influenza vaccination policies targeted at HCP can be integrated in Israel in non-coercive ways. HCP influenza vaccination uptake in Israel has plenty of room for improvement. Public health professionals, healthcare setting administrators, and HCP alike can learn from the successes of American mandatory influenza vaccination policies for HCP when rethinking regulation and practices in Israel.

## Data Availability

The dataset leading to the article’s conclusions is included within the article itself.

## Abbreviations

BJC: Barnes-Jewish-Christian Healthcare
CDC: Centers for Disease Control and Prevention
HCP: Healthcare Personnel
SHEA: The Society for Healthcare Epidemiologists of America
